# Secretory LGALS3BP exacerbates sepsis-associated liver dysfunction by activating inflammasome-mediated pyroptosis

**DOI:** 10.1038/s41420-026-03198-5

**Published:** 2026-06-23

**Authors:** Jun-Eul Hwang, Hyun-Jeong Shim, Mi-Ra Park, Young-Kook Kim, Woo-Kyun Bae, Sang-Hee Cho, Ik-Joo Chung, Eun-Gene Sun

**Affiliations:** 1 Department of Internal Medicine, Division of Hematology and Oncology, Chonnam National University Medical School and Hwasun Hospital, Hwasun, Jeollanam-do Republic of Korea; 2 Department of Biochemistry, Chonnam National University Medical School, Hwasun, Jeollanam-do Republic of Korea; 3 National Immunotherapy Innovation Center, Chonnam National University Medical School, Hwasun, Jeollanam-do Republic of Korea

**Keywords:** Diseases, Cell death

## Abstract

Sepsis is a life-threatening syndrome characterized by a dysregulated host response to infection, leading to multiorgan dysfunction. Inflammasome activation and pyroptosis are key mechanisms driving sepsis-associated organ dysfunction. However, it remains unclear whether circulating biomarkers actively contribute to disease progression. Although galectin-3-binding protein (LGALS3BP) has been proposed as a diagnostic and prognostic marker of sepsis, its functional role in sepsis-associated organ dysfunction remains undefined. In this study, the dynamics and function of LGALS3BP in sepsis were investigated using cecal ligation and puncture (CLP) and lipopolysaccharide (LPS)-induced mouse models. Plasma LGALS3BP levels were markedly elevated during sepsis and were accompanied by increased LGALS3BP expression in multiple organs, particularly in the liver. Hepatocyte-specific LGALS3BP overexpression exacerbated sepsis-associated liver injury, as evidenced by elevated serum alanine aminotransferase and aspartate aminotransferase levels, increased hepatocyte death, and reduced survival. Transcriptomic profiling of septic livers revealed that LGALS3BP overexpression markedly enriched cytokine signaling, inflammatory response gene sets, and the pyroptosis pathway, indicating dysregulated immune responses and an injury-driven transcriptional program during sepsis. RNA sequencing and in vitro experiments using primary hepatocytes treated with recombinant LGALS3BP, which mimics secreted LGALS3BP, demonstrated that recombinant LGALS3BP increased pyroptosis-associated gene signatures but was not sufficient to induce pyroptotic cell death. Instead, recombinant LGALS3BP promoted pyroptotic cell death only in the presence of septic stimuli. Mechanistically, under septic conditions, secretory LGALS3BP activated the TLR2–IRF3–NF-κB signaling axis, leading to NLRP3 inflammasome activation, cleavage of caspase-1 and gasdermin D, and increased IL-1β and IL-18 release, thereby promoting pyroptotic cell death. Importantly, antibody-mediated neutralization of secretory LGALS3BP suppressed TLR2–IRF3–NF-κB signaling and concomitantly attenuated inflammasome activation and pyroptosis in hepatocytes. Collectively, these findings identify LGALS3BP as a key pathogenic driver of sepsis-associated liver dysfunction through pyroptosis-mediated hepatocyte injury. Therapeutic targeting of LGALS3BP may therefore represent a promising strategy to mitigate sepsis-related liver failure beyond its proposed role as a circulating biomarker.

## Introduction

Sepsis is a life-threatening syndrome of multiple organ dysfunction caused by a dysregulated host response to infection and is characterized by an excessive systemic inflammatory response (systemic inflammatory response syndrome; SIRS) that leads to tissue injury and organ failure [1, 2]. Despite advances in critical care, sepsis remains one of the leading causes of death worldwide, accounting for nearly 20% of global mortality [3]. Current diagnostic strategies rely extensively on clinical scoring systems, including the Sequential Organ Failure Assessment (SOFA) and its simplified version, the quick SOFA (qSOFA) [1]. While these systems aid in evaluating disease severity and predicting outcomes, their diagnostic accuracy is limited by poor sensitivity and specificity, particularly in the early stages of sepsis [4]. Conventional biomarkers, such as procalcitonin and C-reactive protein, are widely used as supportive biomarkers but are also elevated in various infectious and noninfectious inflammatory conditions, further limiting their diagnostic utility [5–7]. Notably, the lack of reliable early biomarkers and actionable molecular targets directly contributes to delayed intervention and poor clinical outcomes. Similarly, therapeutic options remain largely supportive, and effective targeted treatments that modulate the host response are still lacking [8, 9]. These limitations underscore the urgent need to identify novel molecular biomarkers that not only reflect disease severity but also inform mechanism-based therapeutic strategies to facilitate early diagnosis and more precise interventions in sepsis [10].

Galectin-3 binding protein (LGALS3BP, also known as 90 K or Mac-2 binding protein) is a heavily glycosylated, secreted, multifunctional protein that regulates immune responses, cell adhesion, and interactions with the extracellular matrix [11–14]. LGALS3BP has been implicated in a broad spectrum of human diseases, including inflammatory bowel disease, liver fibrosis, autoimmune disorders, and various malignancies [12, 15–24]. Recent studies have identified LGALS3BP as a promising biomarker for infectious and inflammatory diseases, particularly sepsis and COVID-19 [25–29]. Serum LGALS3BP levels correlate positively with clinical severity indices such as the Acute Physiology and Chronic Health Evaluation II (APACHE II) and Sequential Organ Failure Assessment (SOFA) scores, supporting its potential utility in evaluating disease progression. Moreover, serum LGALS3BP levels are markedly higher in nonsurvivors than in survivors, underscoring its prognostic value for mortality [25, 26]. Despite these strong clinical associations, the functional relevance of LGALS3BP in the molecular pathogenesis of sepsis has not been elucidated. The primary cause of mortality in sepsis is multiple organ failure, which results from overwhelming systemic inflammation, including cytokine storms and dysregulated forms of cell death [1, 2]. Among these mechanisms, inflammasome activation and pyroptosis are central drivers of organ dysfunction in sepsis [30, 31]. Pyroptosis is an inflammatory form of programmed cell death that is mediated by the inflammasome complex. Inflammasomes function as cytosolic pattern recognition platforms that detect pathogen-associated molecular patterns (PAMPs) and damage-associated molecular patterns (DAMPs), triggering caspase-1 activation, cleavage of gasdermin D, and the release of interleukin (IL)-1β and IL-18 [30, 32–35]. While these processes contribute to antimicrobial host defense, their excessive or sustained activation leads to hepatocellular injury, tissue destruction, and systemic organ dysfunction, positioning pyroptosis as a key pathogenic process rather than a mere bystander response.

Given the emerging evidence linking LGALS3BP to sepsis outcomes and the established role of pyroptosis in organ failure, it remains uncertain whether secretory LGALS3BP merely reflects disease severity or actively participates in the molecular mechanisms underlying inflammatory cell death during sepsis. In particular, the potential role of elevated secretory LGALS3BP in hepatocellular pyroptosis and its contribution to sepsis-related liver dysfunction has not yet been investigated. Addressing this knowledge gap may provide mechanistic insights into the pathophysiological role of LGALS3BP beyond its proposed function as a circulating biomarker and may uncover new opportunities for mechanism-based targeted therapeutic intervention.

## Results

### Lgals3bp expression and secretion are increased in the septic mouse model

**Fig. 1:**
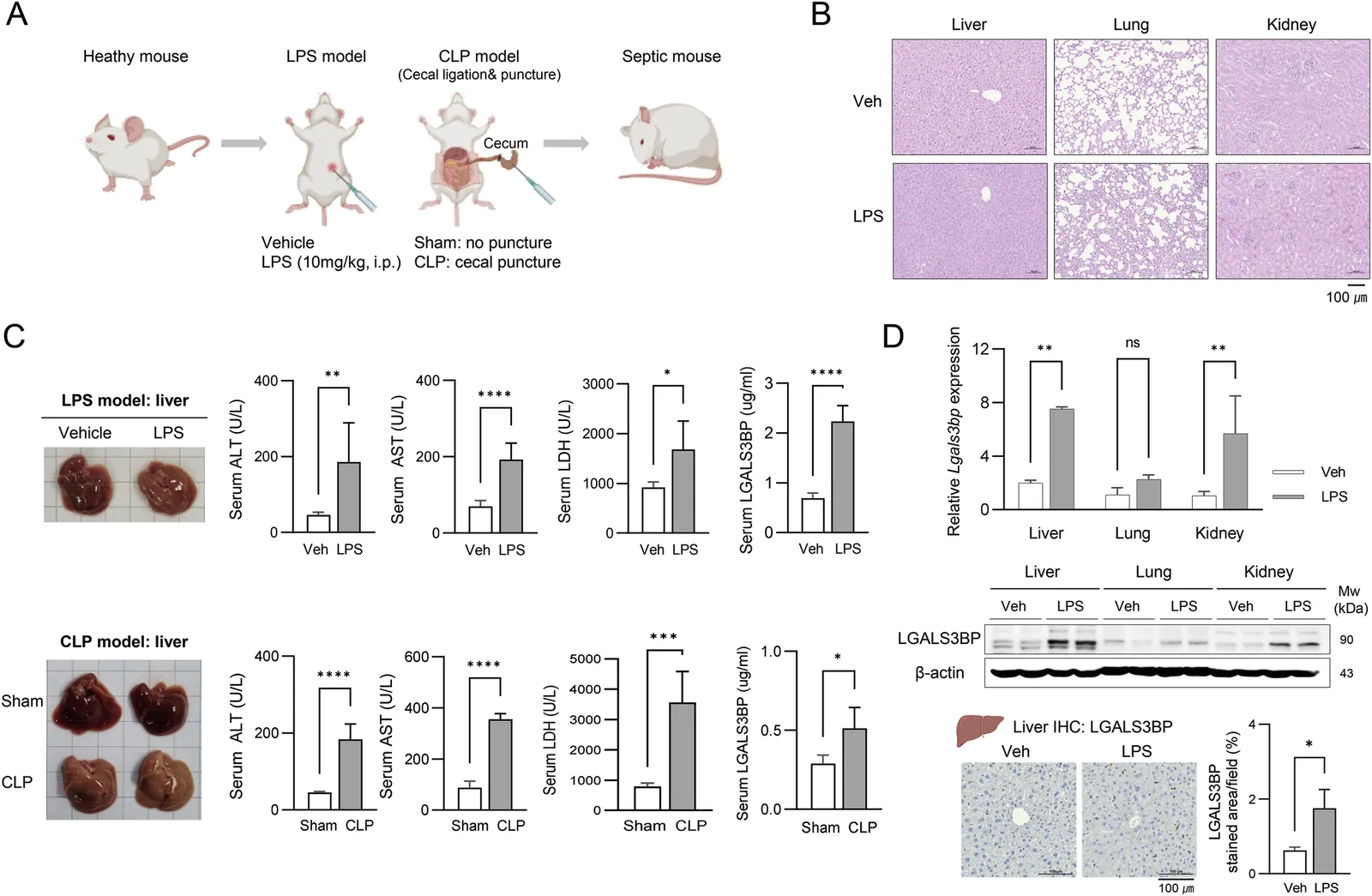
Lgals3bp expression and secretion are upregulated in murine models of sepsis. **A** Schematic overview of the lipopolysaccharide (LPS, 10 mg/kg, i.p.)-induced endotoxemia model and the cecal ligation and puncture (CLP)-induced polymicrobial sepsis model used in this study. Vehicle-treated mice and sham-operated mice served as controls for the LPS and CLP models, respectively. **B** Representative hematoxylin and eosin (H&E)-stained sections of liver, lung, and kidney tissues from vehicle- and LPS-treated mice, illustrating inflammatory cell infiltration and disruption of tissue architecture in septic organs (scale bar = 100 µm). **C** Representative gross images of livers from control and septic mice. Serum alanine aminotransferase (ALT), aspartate aminotransferase (AST), and lactate dehydrogenase (LDH) levels, as well as circulating LGALS3BP concentrations, were measured via serum biochemistry analysis and ELISA in sepsis models (*n* = 6 per group). **D** Quantitative RT‒PCR and immunoblot analyses showing increased Lgals3bp mRNA and protein expression in septic mice, with the most pronounced upregulation in the liver compared with the lung and kidney. Immunohistochemical analysis further confirmed a marked increase in LGALS3BP expression throughout the liver in septic mice (scale bar = 100 µm). The data are presented as the means ± SEMs. Statistical significance was determined via two-way ANOVA followed by Tukey’s multiple comparisons test and an unpaired two-tailed Student’s t test. ns, not significant, *p < 0.05, **p < 0.01, ***p < 0.001, ****p < 0.0001. A schematic illustration was created via BioRender.com.

To evaluate the changes in LGALS3BP expression during sepsis, we first established two mouse models: an LPS-induced sepsis model and a CLP-induced sepsis model (Fig. 1A). Septic mice exhibited extensive inflammatory cell infiltration and severe disruption of tissue architecture across multiple organs, which was consistent with systemic inflammatory injury (Fig. 1B). Similarly, the serum levels of ALT, AST, and LDH were significantly increased, indicating the development of sepsis and sepsis-associated liver dysfunction (SALD). Under these conditions, circulating LGALS3BP levels were notably elevated (Fig. 1C). In parallel, LGALS3BP expression was strongly induced at both the mRNA and protein levels in multiple organs, with the most pronounced upregulation observed in the liver. Consistent with these results, immunohistochemical analysis confirmed widespread increases in LGALS3BP expression throughout hepatic tissue (Fig. 1D). Overall, these findings demonstrate that LGALS3BP expression and systemic secretion are significantly upregulated during sepsis, suggesting a potential role for LGALS3BP in the pathophysiology of sepsis and SALD.

### Hepatic LGALS3BP overexpression exacerbates sepsis severity and liver injury

**Fig. 2:**
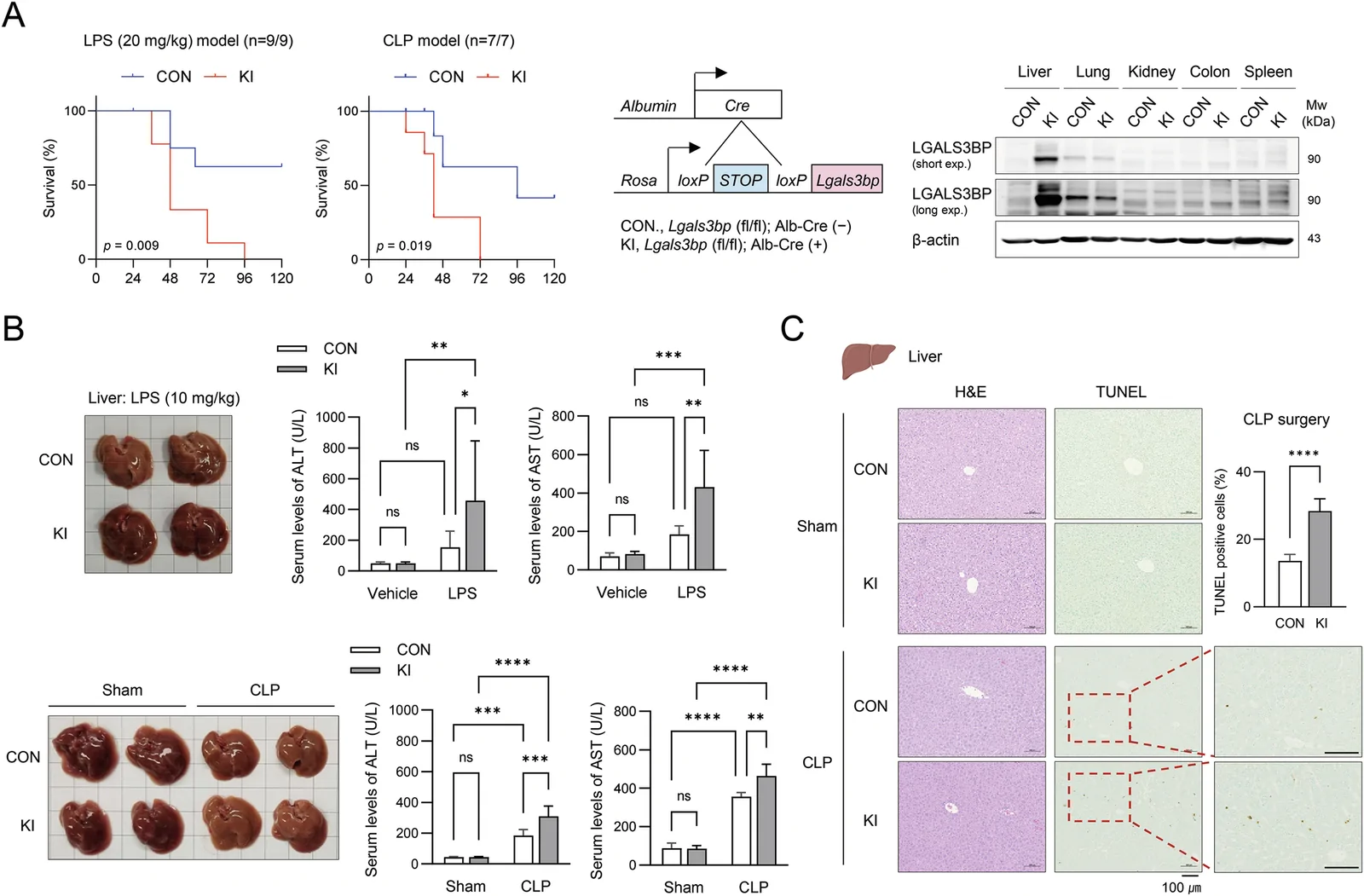
Hepatic LGALS3BP overexpression exacerbates sepsis severity and liver injury. **A** Left: Kaplan‒Meier survival curves for control (CON) and hepatocyte-specific Lgals3bp knock-in (KI) mice after lipopolysaccharide (LPS; 20 mg/kg, i.p.; *n* = 9 per group) or cecal ligation and puncture (CLP)-induced sepsis (*n* = 7 per group). KI mice had significantly lower survival rates than CON mice. Middle: Schematic diagram illustrating the generation of hepatocyte-specific Lgals3bp knock-in (KI) mice using a Cre-inducible LoxP-STOP-LoxP (LSL) system, in which Alb-Cre-mediated excision of the STOP cassette enables hepatocyte-specific overexpression of Lgals3bp. Right: Immunoblot analysis confirmed the specific and robust overexpression of LGALS3BP in the livers of KI mice relative to those of CON mice. **B** Representative gross images of livers from CON and KI mice under LPS- or CLP-induced sepsis conditions. Serum levels of alanine aminotransferase (ALT) and aspartate aminotransferase (AST) were measured by serum biochemistry, which revealed increased liver injury in the KI mice (*n* = 6 per group). **C** Representative hematoxylin and eosin (H&E)-stained and terminal deoxynucleotidyl transferase dUTP nick-end labeling (TUNEL)-stained liver sections from CON and KI mice after CLP surgery, showing severe disruption of liver architecture, inflammatory cell infiltration, and greater hepatic injury in KI mice. Scale bar = 100 µm. The data are shown as the means ± SEMs. Statistical analyses included two-way ANOVA with Tukey’s multiple comparisons for panel **B**, an unpaired two-tailed Student’s t test for panel **C**, and the log-rank (Mantel‒Cox) test for panel **A**. ns, not significant; *p < 0.05, **p < 0.01, ***p < 0.001, ****p < 0.0001.

To investigate the role of LGALS3BP in sepsis pathogenesis, we generated hepatocyte-specific Lgals3bp knock-in (KI) mice. Compared with those in control mice, LGALS3BP levels were significantly increased in the livers of KI mice, and circulating levels of LGALS3BP were also elevated (Fig. 2A and Supplementary Fig. 1A). Compared with control mice, KI mice exhibited significantly lower survival rates under both LPS- and CLP-induced septic conditions, indicating aggravated severity of sepsis associated with hepatic LGALS3BP overexpression (Fig. 2A). Consistently, the serum ALT and AST levels were significantly elevated in the KI mice during sepsis, reflecting more severe liver damage (Fig. 2B and Supplementary Fig. 1B). Histopathological analysis further revealed extensive hepatic damage, pronounced disruption of hepatic architecture, and increased hepatocellular death in KI mice compared with controls under septic conditions (Fig. 2C). Overall, these results indicate that hepatic LGALS3BP overexpression exacerbates sepsis-associated liver injury and mortality, supporting a pathogenic role of LGALS3BP in the development of SALD. Importantly, these in vivo findings suggest that elevated LGALS3BP functions as an active disease-associated factor rather than merely serving as a passive biomarker during sepsis.

### Hepatic Lgals3bp overexpression reprograms the septic liver transcriptome toward inflammatory, LPS-responsive, and pyroptosis-associated pathways

**Fig. 3:**
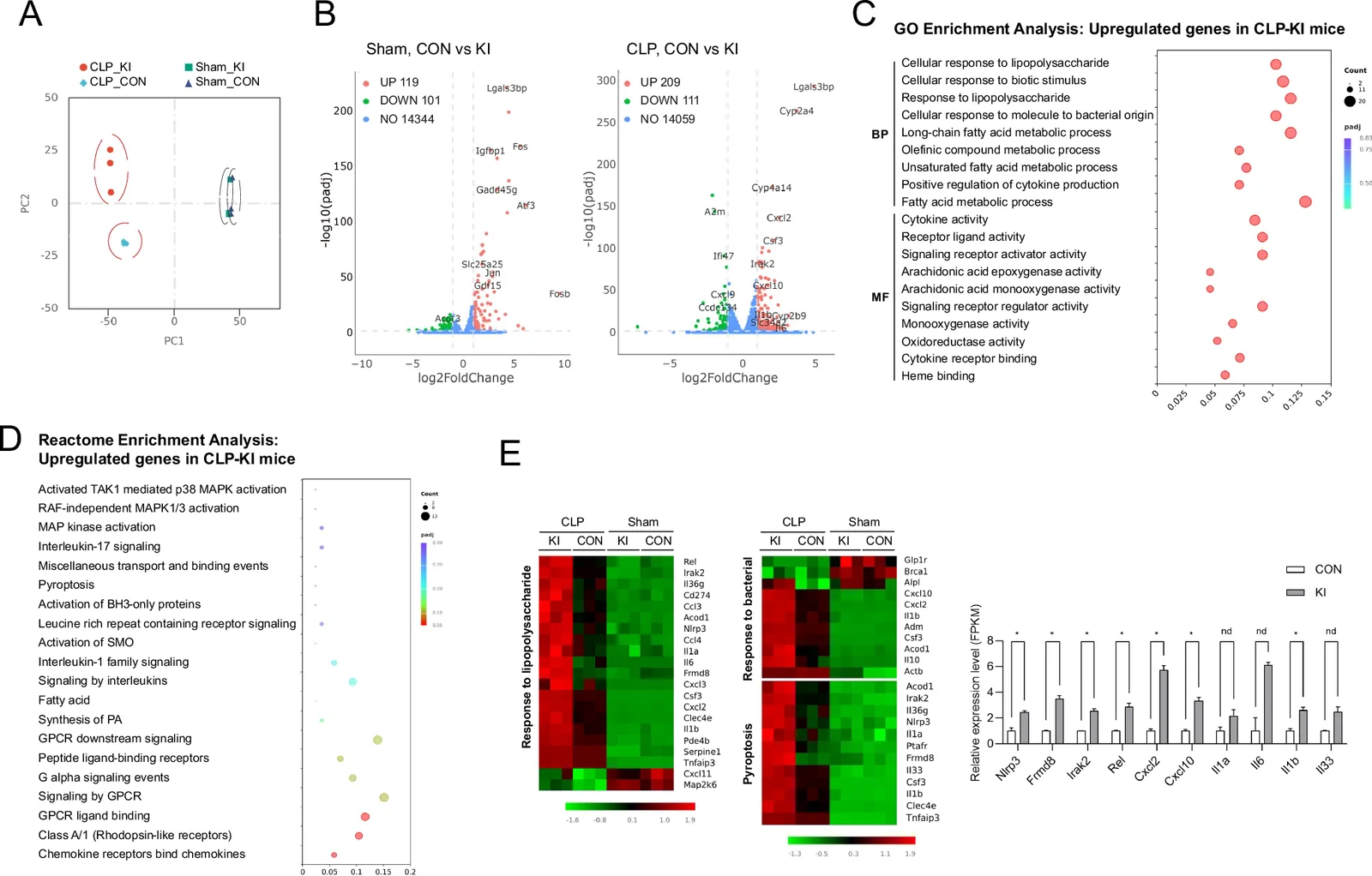
Hepatic LGALS3BP overexpression exacerbates sepsis-induced transcriptional reprogramming toward inflammatory and pyroptosis-associated pathways in the liver. **A** Principal component analysis (PCA) of RNA-seq data from the livers of control (CON) and Lgals3bp knock-in (KI) mice under sham and CLP conditions (*n* = 3 per group), showing distinct clustering according to genotype and sepsis status. **B** Volcano plot depicting differentially expressed genes (DEGs) between KI and CON livers under sham or CLP conditions. DEGs were defined as genes with a fold change> 2 and an adjusted *p* value < 0.05 in KI livers compared with CON livers. Red and green dots indicate significantly upregulated and downregulated genes, respectively. **C, D** Gene Ontology (GO) and Reactome pathway enrichment analyses of 209 genes whose expression was upregulated in CLP-KI livers compared with that in CLP-CON livers revealed significant enrichment of genes related to inflammatory signaling, the bacterial response, and pyroptosis-related pathways. **E** Left, heatmap illustrating the coordinated upregulation of inflammasome-, bacterial response-, and pyroptosis-related genes in KI mice compared with CON mice under CLP-induced sepsis. Right, RNA-seq-based normalized expression levels of representative pyroptosis-related genes identified as significantly upregulated in CLP-KI livers by differential expression analysis. The data are presented as normalized transcript counts. Benjamini‒Hochberg correction was applied for multiple testing.

To explore how LGALS3BP affects SALD, we performed RNA sequencing on liver tissues from CON and Lgals3bp KI mice under both sham and CLP conditions. Principal component analysis (PCA) revealed that the CON and KI samples clustered closely under sham conditions, indicating similar baseline transcriptional states. However, after CLP challenge, the two groups separated clearly, demonstrating that LGALS3BP overexpression caused distinct transcriptional reprogramming, mainly under septic stress (Fig. 3A and Supplementary Fig. 2A). RNA-seq analysis of CLP-induced septic livers revealed 209 upregulated genes and 111 downregulated genes in KI mice compared with CON mice (fold change >2, adjusted *p* value < 0.05) (Fig. 3B). Pathway analysis revealed that genes upregulated in CLP-KI livers were involved mainly in inflammatory and innate immune pathways, including responses to LPS and bacterial stimuli, cytokine‒cytokine receptor interactions, and positive regulation of cytokine production. From the perspective of liver injury, the pyroptosis-related gene set was significantly changed in CLP-KI livers (Fig. 3C–E). Gene set enrichment analysis (GSEA) further confirmed the significant enrichment of interleukin-1 and interleukin-17 signaling, the TLR‒MYD88 cascade, and NOD signaling pathways in the livers of KI mice during sepsis (Supplementary Fig. 2B). Heatmap analysis highlighted the coordinated upregulation of genes related to the response to LPS and bacteria, as well as pyroptosis-related genes, in KI mice (Fig. 3E). Overall, these results suggest that hepatic LGALS3BP overexpression amplifies sepsis-induced transcriptional programs associated with the inflammatory response and pyroptosis, supporting its role as a molecular amplifier of innate immune signaling and hepatocellular injury during sepsis.

### Recombinant LGALS3BP treatment directly reprograms hepatocyte transcription toward inflammasome activation and pyroptosis

**Fig. 4:**
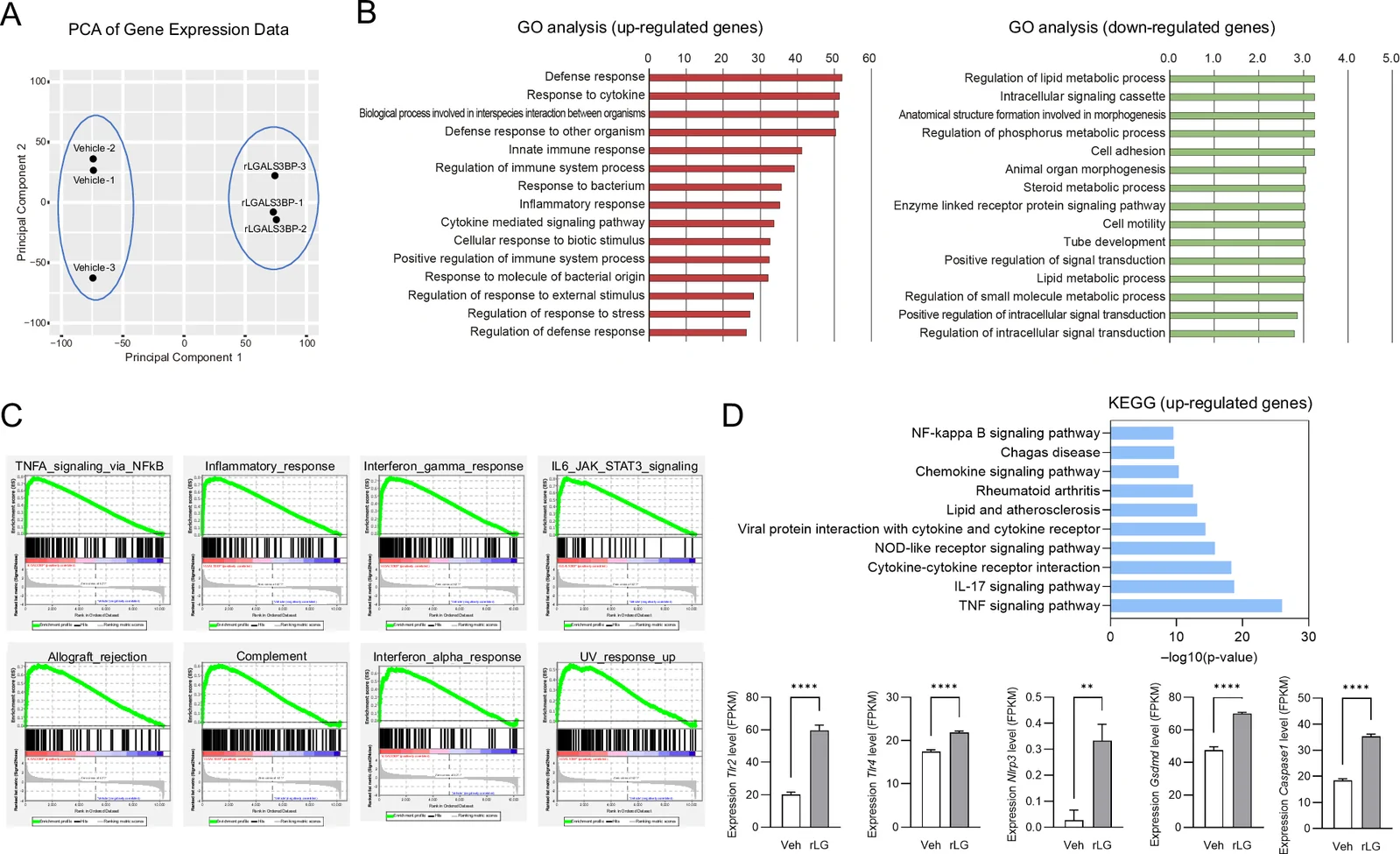
Recombinant LGALS3BP directly induces transcriptional reprogramming toward inflammation and pyroptosis. **A** Principal component analysis (PCA) of RNA-seq data from Hepa1c1c-7 cells treated with vehicle or recombinant LGALS3BP (rLGALS3BP) revealed distinct transcriptional clustering between groups, indicating broad transcriptomic reprogramming following LGALS3BP stimulation (*n* = 3 per group). **B** Gene Ontology (GO) enrichment analysis of upregulated (red) and downregulated (green) genes in rLGALS3BP-treated cells compared with vehicle-treated cells. **C** Gene set enrichment analysis (GSEA) revealed significant enrichment of inflammatory and immune activation pathways, including TNF-α/NF-κB, IL-6–JAK–STAT3 signaling, the interferon response, complement activation, and allograft rejection signatures, in rLGALS3BP-treated cells. **D** KEGG pathway enrichment analysis and RNA-seq-based normalized expression levels of representative genes related to Toll-like receptor (TLR) signaling and pyroptosis, including Nlrp3, Caspase-1, and Gsdmd. The data are shown as normalized transcript counts. Benjamini‒Hochberg correction was used for multiple testing. rLG; recombinant LGALS3BP.

To mechanistically validate the inflammatory and pyroptosis-related signatures observed in vivo, we examined the direct effect of secreted LGALS3BP via recombinant protein stimulation. To this end, we reanalyzed a previously published RNA-seq dataset (Cancer Communications, 2024), which was originally generated for this study: “Galectin-3 binding protein (LGALS3BP) depletion attenuates hepatic fibrosis by reducing transforming growth factor-β1 (TGF-β1) availability and inhibits hepatocarcinogenesis” [20]. The dataset was reprocessed via the same bioinformatics pipeline to assess transcriptional responses to recombinant LGALS3BP (rLGALS3BP) stimulation, which mimics exposure to secretory LGALS3BP. Principal component analysis (PCA) revealed clear transcriptional differences between the two groups (Fig. 4A). Gene Ontology (GO) and Kyoto Encyclopedia of Genes and Genomes (KEGG) enrichment analyses revealed that rLGALS3BP treatment was associated with the activation of inflammatory and innate immune pathways, including defense responses, cytokine signaling, and NOD-like receptor signaling related to inflammasome activation and pyroptosis. Conversely, genes involved in lipid and metabolic processes were significantly downregulated (Fig. 4B, D). Gene set enrichment analysis (GSEA) further confirmed the activation of pathways such as the TNF-α/NF-κB, IL-6–JAK–STAT3, interferon response, complement, and allograft rejection pathways, underscoring the immune-activating capacity of LGALS3BP (Fig. 4C). Differential expression analysis revealed the coordinated upregulation of key genes involved in Toll-like receptor (TLR) signaling and pyroptosis, such as Tlr2, Nlrp3, Caspase-1, and Gsdmd (Fig. 4D). Overall, these findings suggest that secretory LGALS3BP directly reprograms hepatocyte transcription toward inflammatory and pyroptosis-related programs, supporting a role for LGALS3BP as an amplifier of innate immune activation and hepatocellular injury under septic conditions.

### Secretory LGALS3BP promotes NLRP3 inflammasome activation and pyroptotic cell death in hepatocytes

**Fig. 5:**
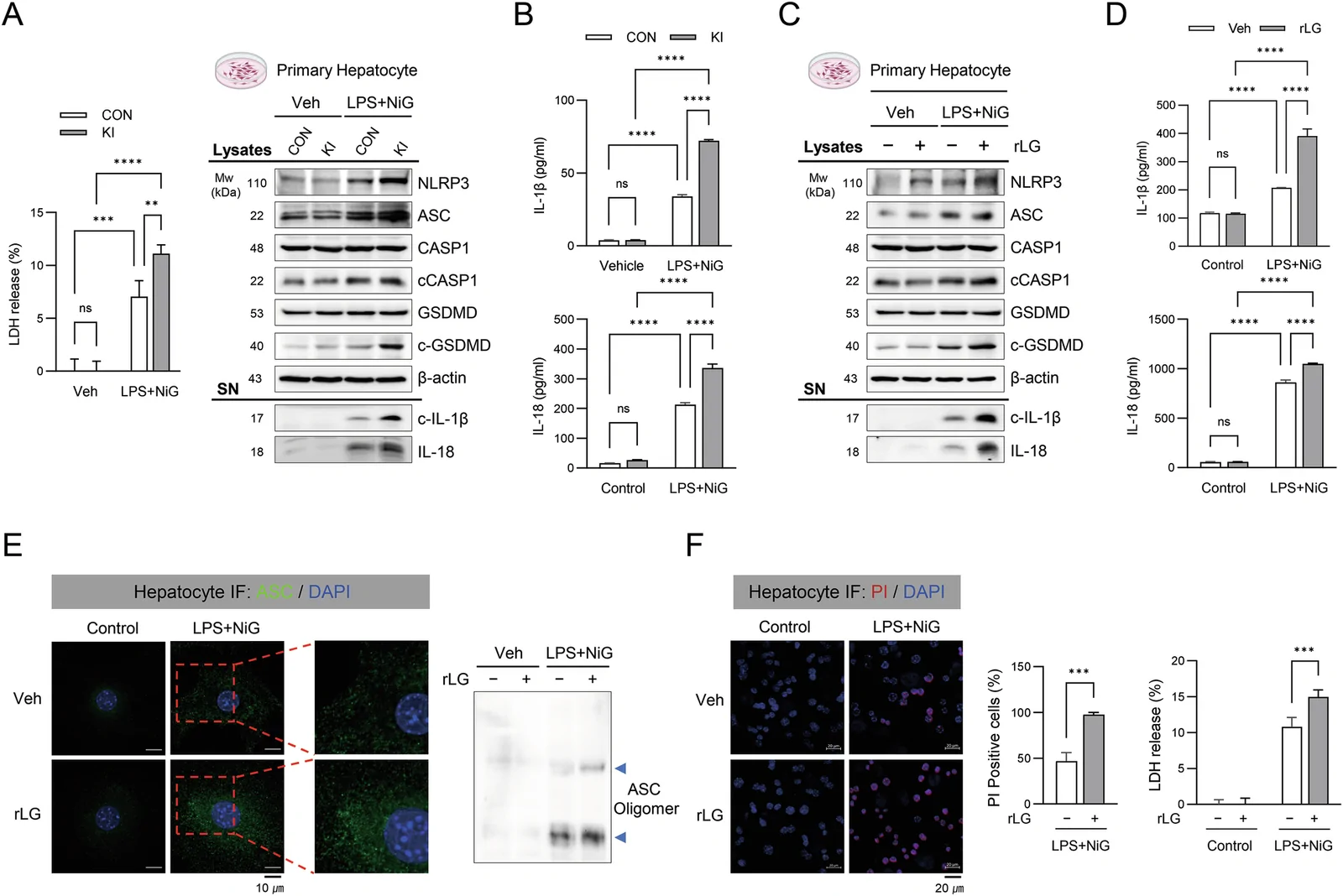
Secretory LGALS3BP promotes NLRP3 inflammasome activation and pyroptotic cell death in hepatocytes. **A, B** Primary hepatocytes were isolated from control (CON) and Lgals3bp knock-in (KI) mice, primed with lipopolysaccharide (LPS; 0.5 μg/mL, 6 h) and then stimulated with nigericin (NiG; 15 μM, 0.5 h). **A** Cytotoxicity was assessed by a lactate dehydrogenase (LDH) release assay. Immunoblot analysis of NLRP3 inflammasome- and pyroptosis-related proteins, including NLRP3, ASC, cleaved caspase-1, cleaved gasdermin **D**, cleaved IL-1β, and IL-18. **B** Enzyme-linked immunosorbent assay (ELISA) of cleaved IL-1β and IL-18 in culture supernatants from CON and KI primary hepatocytes following LPS and nigericin stimulation. Primary hepatocytes from wild-type mice were treated with recombinant LGALS3BP (rLGALS3BP; 1 μg/mL) to mimic secretory LGALS3BP exposure, followed by stimulation with LPS and nigericin. **C** Immunoblot analysis of NLRP3 inflammasome- and pyroptosis-related signaling components. **D** ELISA-based quantification of cleaved IL-1β and IL-18 release in culture supernatants from rLGALS3BP-treated hepatocytes stimulated with LPS and nigericin. **E** Immunofluorescence staining and ASC oligomerization assays demonstrating enhanced ASC speck formation in rLGALS3BP-treated hepatocytes, indicating NLRP3 inflammasome assembly and activation. **F** Immunofluorescence analysis of propidium iodide (PI) uptake and LDH release assays showing increased pyroptotic membrane rupture and cell death in rLGALS3BP-treated hepatocytes after LPS and nigericin stimulation. The data are presented as the means ± SEMs. Statistical analyses were performed via an unpaired two-tailed Student’s t test or two-way ANOVA followed by Tukey’s multiple comparisons test, as appropriate. ns, not significant; **p < 0.01; ***p < 0.001; ****p < 0.0001. Veh vehicle, rLG recombinant LGALS3BP, LPS lipopolysaccharide, NiG nigericin, SN supernatant.

To determine whether LGALS3BP contributes to inflammasome activation and pyroptosis in hepatocytes, we compared primary hepatocytes from Lgals3bp KI and control mice. KI hepatocytes exhibited higher secretion of LGALS3BP than CON cells (Supplementary Fig. 3A). Upon stimulation with LPS and nigericin to mimic septic-like conditions, compared with control hepatocytes, KI hepatocytes presented significantly increased LDH release (Fig. 5A). Immunoblot analyses revealed increased NLRP3 inflammasome activation and pyroptosis, as evidenced by increased NLRP3 and the cleavage of caspase-1 (cCASP1) and gasdermin D (GSDMD), along with increased secretion of cleaved IL-1β (cIL-1β) and IL-18 (Fig. 5A, B). These findings suggest that hepatocyte-derived LGALS3BP amplifies inflammasome activation and pyroptotic cell death under sepsis-like conditions. To test whether secretory LGALS3BP is sufficient to elicit these effects, wild-type hepatocytes were treated with rLGALS3BP. Importantly, rLGALS3BP alone did not induce pyroptotic cell death in the absence of septic stimuli. Under septic conditions, rLGALS3BP treatment recapitulated the inflammasome activation and pyroptotic cell death responses observed in KI hepatocytes (Fig. 5C, D). Additionally, rLGALS3BP increased ASC speck formation, a hallmark of inflammasome assembly, and enhanced membrane permeability, as shown by propidium iodide uptake and LDH release assays (Fig. 5E, F, and Supplementary Fig. 3B). Overall, these results indicate that secreted LGALS3BP promotes inflammasome activation and pyroptotic cell death, thereby amplifying hepatocellular injury under septic conditions.

### LGALS3BP amplifies NLRP3 inflammasome activation and pyroptotic signaling in the septic liver

**Fig. 6:**
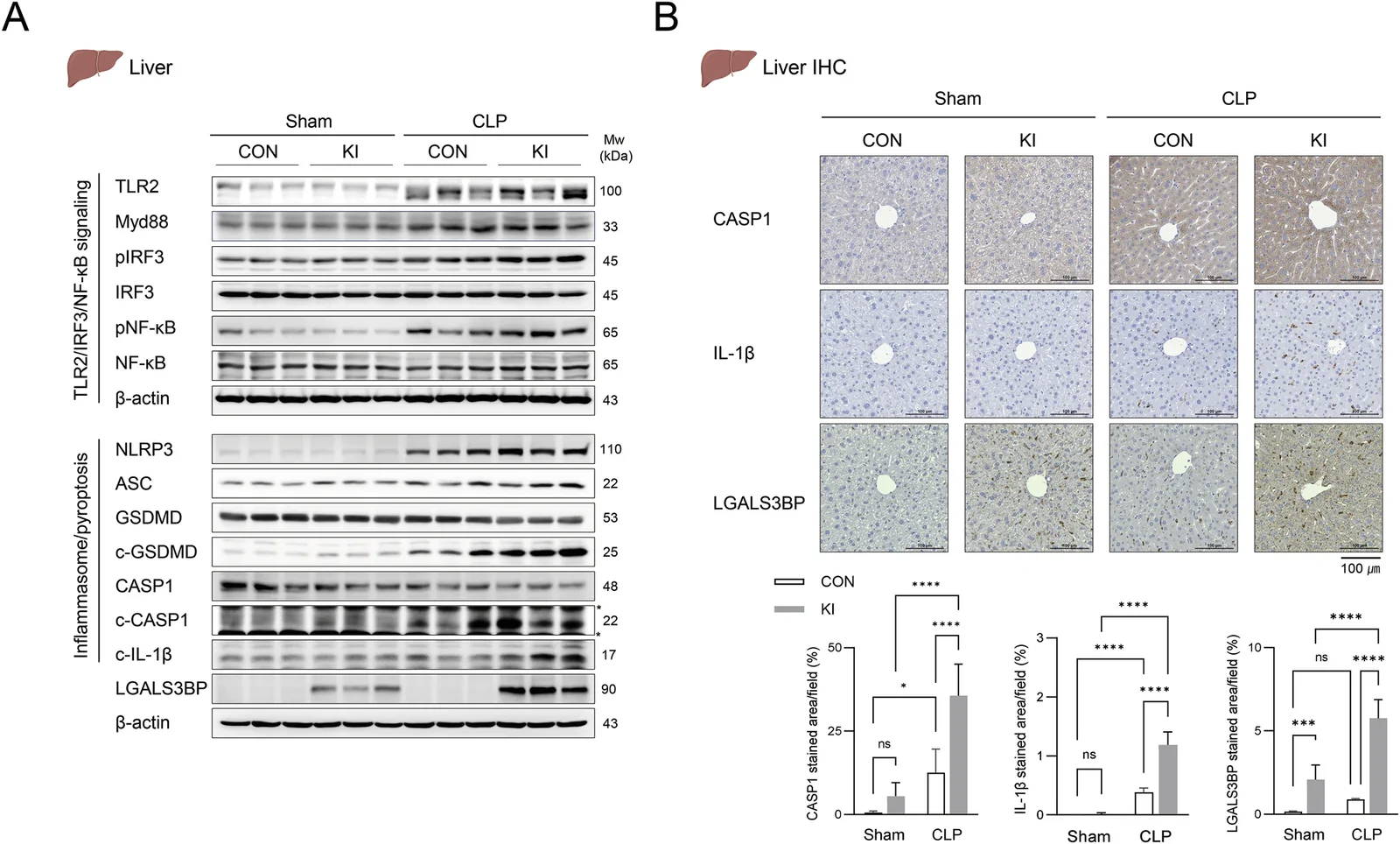
LGALS3BP amplifies TLR2-IRF3-NF-κB signaling and pyroptosis in the septic liver. **A** Immunoblot analysis of liver tissues from Lgals3bp knock-in (KI) and control (CON) mice after sham or CLP surgery. Compared with CON, CLP-induced sepsis increased TLR2 expression and enhanced the phosphorylation of IRF3 and NF-κB p65 in KI livers, accompanied by elevated expression of NLRP3, ASC, cleaved caspase-1, and cleaved gasdermin D. For comparative analysis across four groups, representative samples (*n* = 3 per group) were analyzed within the same blot. An asterisk indicates a nonspecific band. **B** Immunohistochemical staining of liver sections revealed increased CASP1 and IL-1β expression in KI mice compared with CON mice under septic conditions. (scale bar = 100 µm). IHC analysis was performed with *n* = 6 per group, and the positively stained areas were quantitatively evaluated. The data are expressed as the means ± SEMs. Statistical significance was determined via two-way ANOVA followed by Tukey’s multiple comparisons test. ns, not significant; *p < 0.05, **p < 0.01; ***p < 0.001; ****p < 0.0001.

To evaluate whether LGALS3BP enhances inflammasome activation and pyroptosis in vivo, liver tissues from Lgals3bp knock-in (KI) and control (CON) mice were analyzed following sham or CLP surgery. Immunoblot analyses revealed that, compared with CON, CLP-induced sepsis markedly increased the expression of NLRP3 and ASC, as well as the cleavage of caspase-1 and gasdermin D (GSDMD), in the livers of KI mice (Fig. 6A). Consistent with these molecular findings, immunohistochemical staining demonstrated increased immunoreactivity for CASP1 and IL-1β in KI livers under septic conditions (Fig. 6B). Overall, these results suggest that hepatic LGALS3BP overexpression amplifies inflammasome activation and pyroptosis-related liver injury during sepsis.

### LGALS3BP activates the inflammasome and pyroptosis through TLR2-IRF3-NF-κB signaling, which is attenuated by LGALS3BP neutralization

**Fig. 7:**
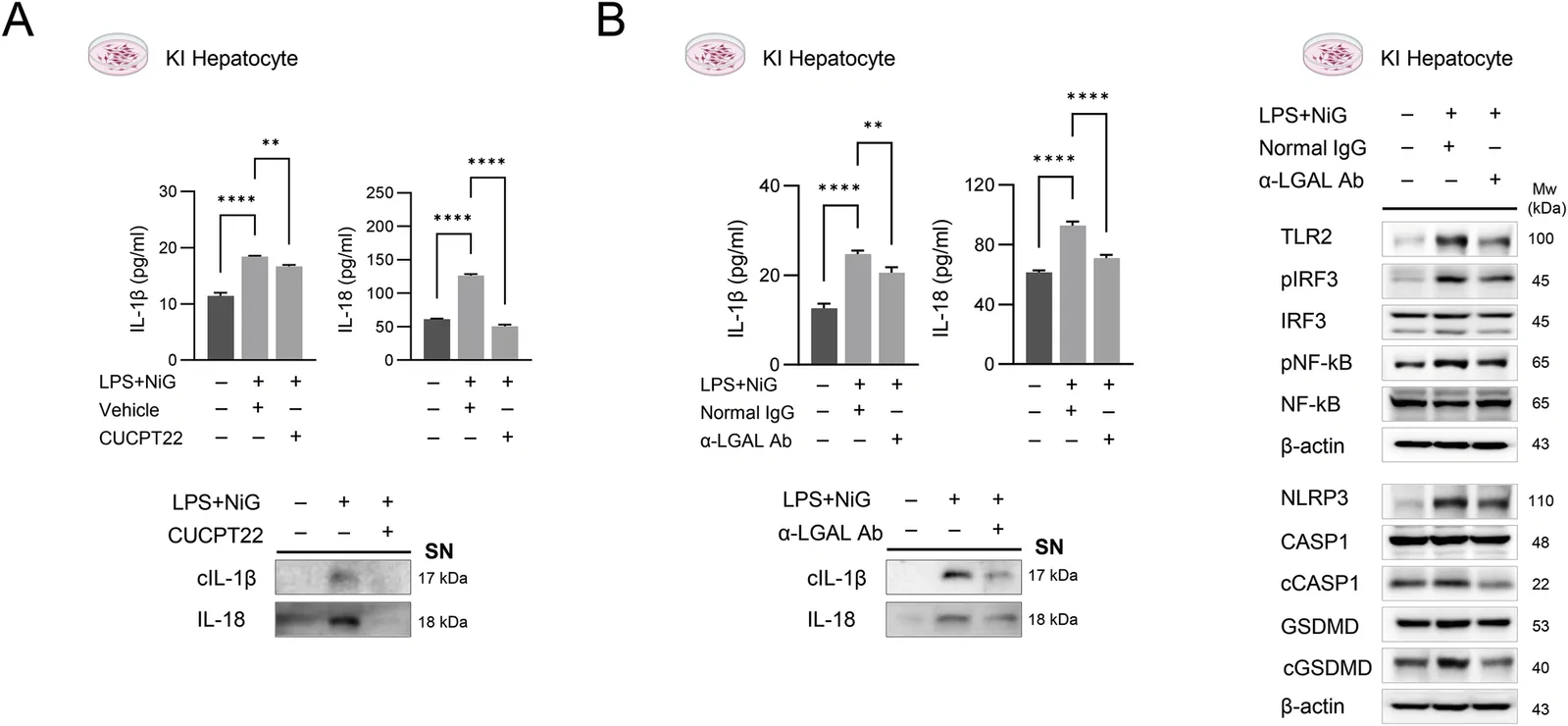
LGALS3BP promotes hepatocyte pyroptosis via the TLR2-IRF3-NF-κB signaling pathway, and this effect is attenuated by LGALS3BP neutralization. **A** Primary hepatocytes isolated from Lgals3bp knock-in (KI) mice were stimulated with LPS plus nigericin in the presence or absence of the TLR2 inhibitor CU-CPT22. The concentrations of IL-1β and IL-18 in the culture supernatants were measured via ELISA (upper panels). Cleaved IL-1β and IL-18 in the supernatants were analyzed by immunoblotting (lower panels). **B** KI hepatocytes were pretreated with a neutralizing anti-LGALS3BP antibody (2.5 μg/mL, 1 h) or control IgG, followed by stimulation with LPS and nigericin. IL-1β and IL-18 levels in the supernatants were measured via ELISA, and the release of cleaved IL-1β and IL-18 was assessed by immunoblotting (left panels). The activation of the TLR2-IRF3-NF-κB signaling pathway and the expression of pyroptosis-related proteins were analyzed by immunoblotting (right panels). The data are presented as the means ± SEMs. Statistical significance was determined via one-way ANOVA followed by Tukey’s post hoc test. **p < 0.01, ****p < 0.0001. CU-CPT22, TLR2 inhibitor.

Toll-like receptors (TLRs) are critical sensors of PAMPs and DAMPs that initiate innate immune responses during sepsis, and they are closely linked to the inflammasome and pyroptosis [36]. LGALS3BP has been reported to activate TLR2 signaling in metabolic inflammation [24], suggesting that LGALS3BP may engage TLR2-dependent pathways under septic conditions. In line with these findings, our transcriptomic analyses revealed significant enrichment of the TLR-related gene set in the KI liver during sepsis (Supplementary Fig. 2B). Based on these observations, we next examined whether LGALS3BP is involved in activating TLR2 signaling and downstream inflammasome responses in vivo. Compared with controls, CLP-induced sepsis led to a notable increase in TLR2 expression and increased phosphorylation of IRF3 and NF-κB p65 in KI livers, accompanied by downstream pyroptosis-related molecular changes (Fig. 6A). To functionally confirm the role of TLR2 signaling, primary hepatocytes isolated from KI mice were stimulated with LPS and nigericin, with or without the TLR2 inhibitor CU-CPT22. Pharmacological inhibition of TLR2 significantly decreased the release of cIL-1β and IL-18, indicating that TLR2 signaling is required for LGALS3BP-associated inflammasome activation and pyroptotic responses. Moreover, neutralizing secreted extracellular LGALS3BP with a specific antibody effectively attenuated TLR2-IRF3-NF-κB signaling and reduced the expression of NLRP3, cCASP1, and cGSDMD, as well as the secretion of cIL-1β and IL-18 in hepatocytes (Fig. 7B). Overall, these findings support a model in which secreted LGALS3BP acts upstream of TLR2 signaling to increase IRF3-NF-κB activation, thereby promoting inflammasome activation and pyroptosis during sepsis. Importantly, antibody-mediated blockade of LGALS3BP effectively suppressed these responses, highlighting its potential as a therapeutic target for SALD.

## Discussion

Sepsis is a life-threatening syndrome characterized by organ dysfunction caused by a dysregulated host response to infection [1, 3, 8]. Accumulating evidence indicates that excessive inflammasome activation and pyroptosis are central mechanisms driving sepsis-associated organ failure, including SALD [30, 31]. Although circulating levels of LGALS3BP are consistently elevated in sepsis and other systemic inflammatory disorders [25, 27, 37], its mechanistic role in sepsis-associated organ failure, particularly in the liver, remains undefined. In the present study, we provide in vivo and mechanistic evidence that LGALS3BP exacerbates hepatic inflammasome activation and pyroptotic injury, thereby aggravating SALD and overall sepsis severity.

Across systemic inflammatory conditions, including sepsis, severe COVID-19, and systemic inflammatory response syndrome (SIRS), circulating LGALS3BP levels are consistently elevated, supporting its potential as a diagnostic biomarker [23, 27, 29]. However, the biological roles of LGALS3BP are context-dependent and controversial. For example, while several studies on COVID-19 have reported no direct antiviral activity of LGALS3BP [27], others have described LGALS3BP as a spike-interacting factor that limits pseudoviral entry, implying a protective role [28]. In sepsis, elevated serum LGALS3BP levels are linked to increased disease severity and worse clinical outcomes, but whether LGALS3BP actively drives disease progression remains unresolved. Our findings directly address this unresolved question by demonstrating that LGALS3BP promotes sepsis progression by activating hepatic inflammasomes and inducing pyroptotic cell death. These results shift LGALS3BP from a passive circulating biomarker to an active contributor to liver injury in sepsis, providing new insights into its pathogenic role in SALD.

Mechanistically, secretory LGALS3BP promotes NLRP3 inflammasome activation and enhances downstream caspase-1 and GSDMD signaling, thereby accelerating pyroptotic injury under septic conditions. Although pyroptosis contributes to pathogen clearance, excessive or dysregulated activation of pyroptosis leads to systemic inflammation and organ failure. Our data indicate that hepatocyte-derived LGALS3BP functions as a paracrine sensitizer, amplifying NLRP3 inflammasome-driven pyroptosis and linking elevated LGALS3BP levels to the progression of sepsis and SALD. Importantly, in vitro RNA sequencing and functional analyses in primary hepatocytes demonstrated that rLGALS3BP increased pyroptosis-associated gene signatures but was insufficient to induce pyroptotic cell death (Figs. 4, 5). In vivo, hepatocyte-specific overexpression of LGALS3BP alone also did not directly cause liver injury in the absence of septic stimuli (Figs. 2, 6A). Instead, LGALS3BP functioned as an amplifier, enhancing pyroptotic cell death and liver injury only under septic conditions (Figs. 2, 5, 6). Together, these findings suggest that LGALS3BP acts as a stimulus-dependent amplifier, worsening pyroptotic injury only when pathogen- or damage-related signals are present, thereby converting hepatocytes into high-gain responders to inflammatory danger cues.

Previous studies have suggested that monocyte-lineage cells are a major source of circulating LGALS3BP in sepsis [27]. Extending these observations, our data demonstrate that LGALS3BP is upregulated across multiple organs during sepsis, with the highest expression observed in the liver. Moreover, hepatocyte-specific LGALS3BP overexpression was sufficient to increase circulating LGALS3BP levels (Fig. 1D and Supplementary Fig. 1), supporting a substantial contribution of organ-derived LGALS3BP to the increase in systemic LGALS3BP abundance. While the present study focused on its contribution to SALD, the extrahepatic and systemic immunological roles of LGALS3BP—particularly its effects on circulating immune cells and distant organs—remain to be elucidated. Future studies are needed to determine whether systemic modulation of LGALS3BP influences multiorgan injury and to evaluate its therapeutic potential in broader septic contexts.

Growing evidence indicates that NLRP3 and IL-6 are emerging therapeutic targets in sepsis [34, 38]. Our findings suggest that LGALS3BP functions upstream of both pathways. In LGALS3BP-KI livers, Nlrp3 and IL-6 expression are significantly induced under septic conditions (Fig. 3E), positioning LGALS3BP at the intersection of two central axes of inflammation and pyroptotic cell death that contribute to sepsis severity. Notably, neutralizing secretory LGALS3BP markedly reduced NLRP3 expression, suppressed caspase-1/GSDMD activation, and attenuated hepatocyte pyroptosis, indicating that LGALS3BP potentiates inflammasome-derived cell death pathways. Collectively, these findings suggest that LGALS3BP may function as an amplifier of pyroptosis and cytokine signaling, thereby intersecting with pathways currently under investigation. Within this framework, LGALS3BP has emerged not only as a diagnostic and prognostic biomarker but also as a potential upstream regulator and a druggable target in SALD.

**Fig. 8:**
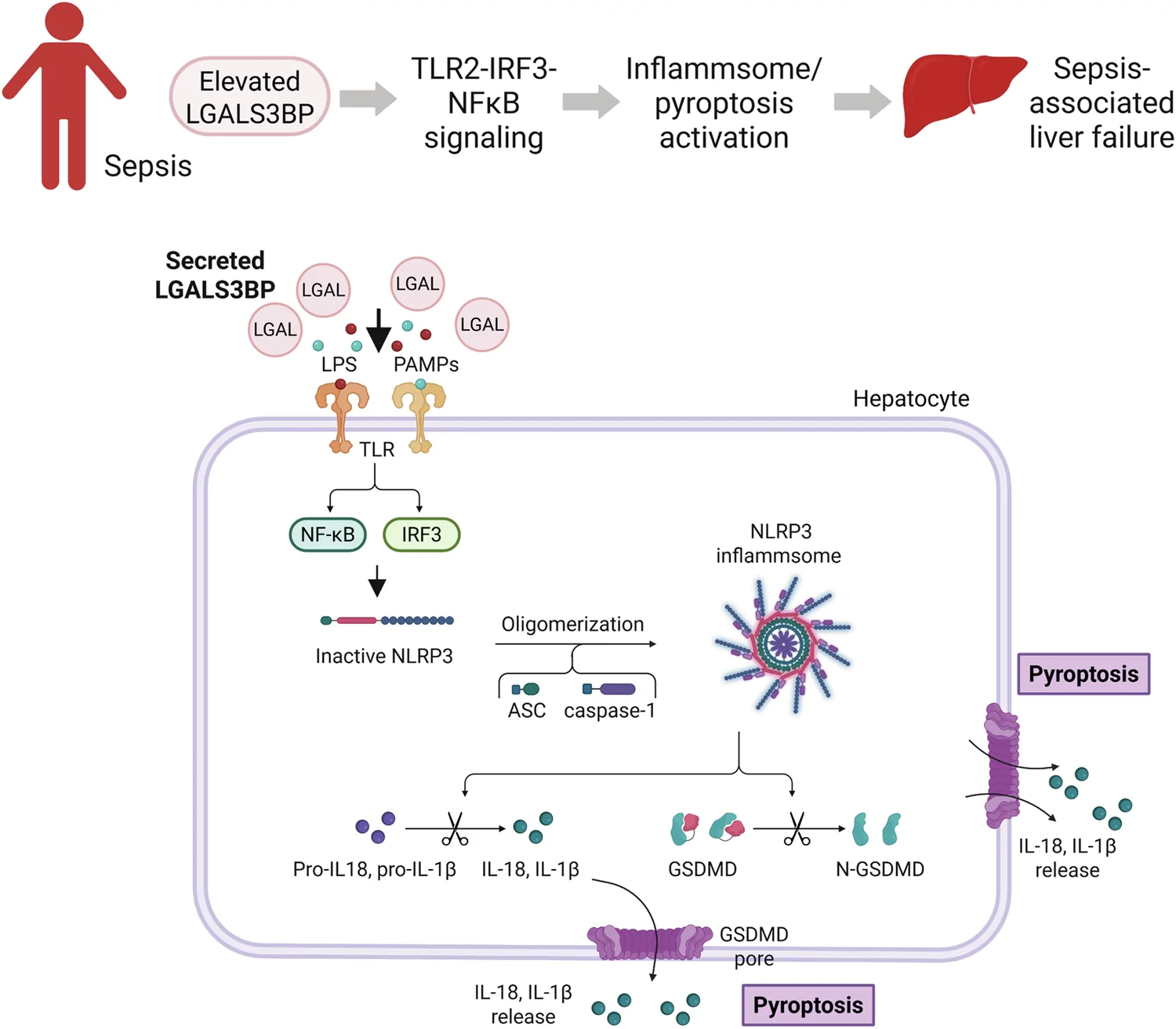
Schematic model illustrating the pathophysiological role of LGALS3BP in sepsis-associated liver dysfunction. During sepsis, circulating and hepatocyte-derived LGALS3BP levels are significantly increased. Secreted LGALS3BP engages the TLR2-IRF3-NF-κB signaling pathway, thereby increasing inflammasome activation and pyroptosis. Activation of the NLRP3 inflammasome leads to ASC oligomerization and caspase-1 activation, resulting in the cleavage of gasdermin D and the maturation of IL-1β and IL-18. The formation of GSDMD pores causes pyroptotic death of hepatocytes and the release of IL-1β and IL-18, which worsen liver injury and accelerate progression toward sepsis-associated liver failure. A schematic illustration was created with BioRender.com.

In conclusion, this study clarifies the pathophysiological role of LGALS3BP beyond its previously recognized use as a diagnostic biomarker in sepsis. Secreted LGALS3BP enhances inflammasome activation and pyroptotic cell death under septic conditions, thereby contributing to the development of SALD (Fig. 8). Importantly, blocking secreted LGALS3BP reduces inflammasome activation and pyroptotic responses, suggesting that LGALS3BP may be a druggable upstream regulator in sepsis and SALD. Taken together with its robust serological detectability, these findings indicate that targeting LGALS3BP therapeutically could be a rational approach to reduce liver injury and improve disease severity in severe sepsis.

## Materials and methods

### Reagents

Lipopolysaccharide (LPS, *Escherichia coli* O111:B4) and D-galactosamine (D-GalN) were purchased from Sigma‒Aldrich (St. Louis, MO, USA). Nigericin was obtained from InvivoGen (San Diego, CA, USA). CU-CPT22 was purchased from MedChemExpress (Monmouth Junction, NJ, USA). Recombinant mouse LGALS3BP (galectin-3-binding protein) was obtained from R&D Systems (Minneapolis, MN, USA). All reagents were of analytical grade and were used according to the manufacturer’s instructions.

### Animals and the septic mouse model

Eight-week-old male C57BL/6 mice were obtained from Orient Bio, Inc. (Seongnam, Korea) and maintained under specific pathogen-free conditions with controlled temperature (22 ± 2 °C), humidity (50 ± 10%), and a 12-h light/dark cycle, with free access to food and water. Hepatocyte-specific Lgals3bp knock-in (KI) mice were generated using a Cre-inducible LoxP-STOP-LoxP (LSL) system. Rosa26-LSL-Lgals3bp homozygous mice were generated by Macrogen (Seoul, Korea), in which LSL cassette followed by the Lgals3bp coding sequence was inserted into the Rosa26 locus [20]. Albumin-Cre transgenic mice were obtained from the Korea Research Institute of Bioscience and Biotechnology (KRIBB, Daejeon, Korea). To achieve hepatocyte-specific overexpression of Lgals3bp, Rosa26-LSL-Lgals3bp homozygous mice were crossed with Albumin-Cre heterozygous mice to generate littermate control and KI mice. Control mice were Lgals3bp(fl/fl); Alb-Cre (−), whereas hepatocyte-specific knock-in (KI) mice were Lgals3bp(fl/fl); Alb-Cre (+). In KI mice, Cre recombinase driven by the albumin promoter mediates excision of the STOP cassette, resulting in hepatocyte-specific overexpression of Lgals3bp. Hepatocyte-specific Lgals3bp KI mice and their littermate control mice were also male and age-matched (8 weeks old) to ensure consistency across experimental groups. All animal experiments were approved by the Institutional Animal Care and Use Committee (IACUC) of Chonnam National University Medical School (Approval CNU IACUC-H-2023-5) and performed in accordance with institutional and national guidelines for the care and use of laboratory animals. For the LPS-induced sepsis model, LPS (*Escherichia coli* O111:B4, Sigma‒Aldrich, St. Louis, MO, USA) was administered intraperitoneally at 20 mg/kg, and survival was monitored for 120 h. A lower LPS dose (10 mg/kg, i.p.) was used in a separate cohort, and the mice were euthanized at designated time points for tissue collection and molecular analyses to assess sepsis-associated organ dysfunction. For the cecal ligation and puncture (CLP) model, polymicrobial sepsis was induced through midline laparotomy. The distal cecum was ligated and punctured once with a 22-gauge needle and gently pressed to extrude a small amount of feces. The cecum was then returned to the abdominal cavity, and the abdomen was sutured in two layers. Postoperative care included subcutaneous fluid resuscitation and continuous monitoring for clinical signs of sepsis. A sham group was established similarly without a puncture [39].

### Histological and immunohistochemical analyses

Liver and other organ tissues were collected at designated time points after LPS or CLP challenge and fixed in 10% neutral buffered formalin for 24 h. Samples were processed, embedded in paraffin, and sectioned at 4 μm. For histopathological evaluation, the sections were stained with hematoxylin and eosin (H&E) to assess tissue morphology and inflammatory injury across multiple organs. Liver sections were further subjected to terminal deoxynucleotidyl transferase dUTP nick-end labeling (TUNEL) assays to detect cell death and to immunohistochemical (IHC) staining for caspase-1, IL-1β, and LGALS3BP. All histological, IHC, and TUNEL analyses were performed by T&P Bio (Gyeonggi, Korea) via standardized protocols. The stained slides were scanned via an Axio Scan. A Z1 digital slide scanner (Carl Zeiss, Oberkochen, Germany) was used, and quantitative analysis was performed via ZEN imaging software (Zeiss).

### Primary hepatocyte isolation

Primary hepatocytes were isolated from 12-week-old male C57BL/6 mice via a two-step collagenase perfusion method. Briefly, the mice were anesthetized, and the liver was perfused in situ through the portal vein with prewarmed Liver Perfusion Medium (Thermo Fisher Scientific, Cat. #17701038) supplemented with 100 U/mL penicillin–streptomycin, followed by Liver Digest Medium (Thermo Fisher Scientific, Cat. #17703034) at a constant flow rate of 2 mL/min. After perfusion, the digested liver was excised, gently minced in cold hepatocyte wash medium (Thermo Fisher Scientific, Cat. #A1217601), and filtered through a 70 μm nylon cell strainer. The cell suspension was centrifuged at 50 × g for 5 min at 4 °C to collect hepatocytes. To enrich viable hepatocytes, the cell pellet was resuspended in 40% Percoll (Cytiva, Marlborough, MA, USA) and centrifuged at 150–200 × g for 7 min. The resulting hepatocytes were resuspended in DMEM/F-12 (Gibco, Grand Island, NY, USA) supplemented with 10% fetal bovine serum (FBS; HyClone, Logan, UT, USA) and 1% penicillin–streptomycin (Gibco). The cells were seeded at a density of 4 × 10⁵ cells/well onto collagen-coated six-well plates and incubated at 37 °C in a humidified atmosphere with 5% CO_2_. After 4 h, nonadherent cells were removed by gentle washing, and adherent hepatocytes were replenished with fresh maintenance medium.

### Hepatocyte stimulation and treatment

After isolation and stabilization, the cells were stimulated with lipopolysaccharide (LPS, 0.5 μg/mL; *Escherichia coli* O111:B4, Sigma‒Aldrich, St. Louis, MO, USA) for 6 h, followed by nigericin (15 μM; InvivoGen) for 0.5 h to activate inflammasomes and pyroptosis. Recombinant mouse LGALS3BP (R&D Systems, Minneapolis, MN, USA) was added at the indicated concentration 1 h before LPS stimulation, unless otherwise specified. For the inhibitor studies, cells were pretreated with CU-CPT22 (10 μM; MedChemExpress, NJ, USA) for 2 h prior to stimulation. The control cells received equivalent volumes of vehicle (0.1% DMSO).

### Serum biochemistry analysis and enzyme-linked immunosorbent assay (ELISA)

Serum alanine aminotransferase (ALT), aspartate aminotransferase (AST), and lactate dehydrogenase (LDH) levels were measured via an automated blood biochemistry analyzer (Catalyst One; IDEXX Laboratories, Inc., Westbrook, ME, USA). The concentrations of LGALS3BP, interleukin-1β (IL-1β), and IL-18 in the serum and cell culture supernatants were determined via commercial enzyme-linked immunosorbent assay (ELISA) kits specific for mouse LGALS3BP, IL-1β, and IL-18 (BD Biosciences, San Jose, CA, USA) according to the manufacturer’s instructions. All measurements were performed at least in triplicate.

### ASC oligomerization assay

Primary hepatocytes were lysed in TBS buffer (50 mM Tris-HCl, 150 mM NaCl, pH 7.4) containing 1% Triton X-100 and a protease inhibitor cocktail (Thermo Fisher Scientific, Waltham, MA, USA). The lysates were subsequently centrifuged at 6,800 × g for 20 min at 4 °C to separate the Triton X-100–soluble and insoluble fractions. The supernatant (soluble fraction) was collected for immunoblot analysis. The Triton X-100–insoluble pellets were resuspended in TBS buffer and crosslinked with 2 mM disuccinimidyl suberate (DSS; Thermo Fisher Scientific, Cat. #A39267) for 30 min at room temperature (RT). The crosslinked samples were centrifuged again at 6,800 × g for 20 min at 4 °C, and the resulting pellets were dissolved in Laemmli buffer (Bio-Rad, Hercules, CA, USA). The samples were then subjected to 12% SDS–PAGE followed by immunoblotting to detect ASC oligomers.

### Immunofluorescence assay

Primary hepatocytes were fixed with 4% (v/v) paraformaldehyde (PFA; Thermo Fisher Scientific, Waltham, MA, USA) for 10 min at room temperature (RT). After fixation, the cells were permeabilized with 0.1% (v/v) Triton X-100 (Fisher Scientific, Cat. #BP151–500) for 10 min at RT. Following permeabilization, the cells were blocked with 1% bovine serum albumin (BSA; Sigma-Aldrich, St. Louis, MO, USA) containing 0.05% Triton X-100 for 1 h at RT and then incubated with an anti-ASC primary antibody diluted in blocking buffer overnight at 4 °C. After washing three times with phosphate-buffered saline (PBS), the cells were incubated with an Alexa Fluor–conjugated secondary antibody (Invitrogen, Carlsbad, CA, USA) for 2 h at 4 °C. Nuclei were counterstained and mounted with ProLong Gold Antifade Mountant with DAPI (Thermo Fisher Scientific, Cat. #P36931). Fluorescence images were acquired via a fluorescence microscope (Zeiss, Jena, Germany) and processed via ZEN imaging software (Zeiss).

### RNA extraction and quantitative real-time PCR (RT‒qPCR)

Total RNA was extracted via a Ribospin RNA extraction kit (GeneAll Biotechnology, Seoul, Korea) following the manufacturer’s instructions. Complementary DNA (cDNA) was synthesized from total RNA via the GoScript Reverse Transcription System (Promega, Madison, WI, USA). Reverse transcription–quantitative PCR (RT‒qPCR) was conducted via SYBR Green chemistry on a CFX96 Real-Time PCR Detection System (Bio-Rad Laboratories, Hercules, CA, USA). TATA-box binding protein (TBP) served as an internal reference gene for normalizing expression levels. The sequences of primers used in this study were as follows: mLgals3bp forward, 5′-TGCTGGTTCCAGGGACTCAA-3′; reverse, 5′-CCACCGGCCTCTGTAGAAGA-3′; mTbp forward, 5′-ACCCTTCACCAATGACTCCTATG-3′; reverse, 5′-TGACTGCAGCAAATCGCTTGG-3′. Relative gene expression was calculated via the 2^ − ΔΔCT method.

### RNA sequencing analysis

Total RNA (1 µg) was extracted from the liver tissues of sham and CLP-induced septic mice via a Ribospin RNA extraction kit (GeneAll Biotechnology, Seoul, Korea). RNA quality and integrity were evaluated via an Agilent 2100 Bioanalyzer (Agilent Technologies, Santa Clara, CA, USA). The RNA libraries were constructed via the TruSeq RNA Library Prep Kit v2 (Illumina, San Diego, CA, USA) and sequenced on an Illumina NovaSeq platform (Novogen, Singapore) to generate 150-bp paired-end reads. For comparative analysis, RNA-seq data from recombinant LGALS3BP-treated primary hepatocytes were obtained from our previous publication [20] and reanalyzed via the same computational pipeline. The raw sequencing reads were trimmed and quality-filtered before alignment to the mouse reference genome (mm10) via the HISAT2 aligner integrated with feature counts for transcript quantification. Gene expression levels were normalized to fragments per kilobase of transcript per million mapped reads (FPKM). Differentially expressed genes (DEGs) were identified on the basis of the following criteria: Benjamini–Hochberg–adjusted *P* value (FDR) < 0.05, absolute log_2_ fold-change (|log_2_FC | ) ≥ 1, and FPKM ≥ 1. Functional enrichment analyses, including Gene Ontology (GO), KEGG, and Reactome pathway analyses, were performed via the Molecular Signatures Database (MSigDB). All RNA sequencing of the CLP liver samples was performed by Novogen (Singapore).

### Western blot analysis

Proteins were extracted from cultured cells and tissue samples via appropriate lysis buffers: RIPA buffer (Sigma‒Aldrich, Cat. #R0278, St. Louis, MO, USA), protein extraction reagent (Thermo Fisher Scientific, Cat. #78501, Waltham, MA, USA), or T-PER Tissue Protein Extraction Reagent (Thermo Fisher Scientific, Cat. #78510). Equal amounts of total protein were separated by SDS–PAGE and transferred to polyvinylidene fluoride (PVDF) membranes (Millipore, Cat. #IPVH00010, Burlington, MA, USA). After being blocked with SuperBlock T20 blocking buffer (Thermo Fisher Scientific, Cat. #37515), the membranes were incubated overnight at 4 °C with the primary antibodies listed in Supplementary Table 1. Following washing with Tris-buffered saline containing 0.1% Tween-20 (TBST), the membranes were incubated with horseradish peroxidase (HRP)-conjugated secondary antibodies for 1 h at RT. Immunoreactive bands were visualized via an enhanced chemiluminescence detection system (LAS-4000 mini; Fujifilm, Tokyo, Japan). Densitometric quantification of protein bands was performed via Multi Gauge software (version 3.0; Fujifilm).

### Statistical analysis

Statistical analyses were performed via GraphPad Prism software (version 10; GraphPad Software, San Diego, CA, USA). Continuous variables are presented as the means ± standard errors of the means (SEMs), whereas categorical variables are presented as frequencies and percentages. Comparisons between two groups were conducted via an unpaired two-tailed Student’s t-test. For comparisons among multiple groups, one-way analysis of variance (ANOVA) followed by Tukey’s post hoc test was used. For experiments with two independent variables, two-way ANOVA with appropriate multiple-comparison corrections was applied. Survival data were analyzed via the Kaplan–Meier method, and differences between groups were assessed via the log-rank (Mantel–Cox) test. Statistical significance was defined as *p* < 0.05. All experiments were performed at least in triplicate unless otherwise noted.

### Experimental design and reproducibility

Samples were randomly allocated to experimental groups in all in vitro experiments. For in vivo studies, mice were randomly assigned to experimental groups (e.g., CON vs. KI and treatment groups) to minimize selection bias. No additional stratification or exclusion criteria were applied during group allocation. No samples or animals were excluded from the analysis. No predefined exclusion criteria were applied, and all collected data were included in the final analysis.

## Supplementary information

Supplementary information

Supplementary Figure 1

Supplementary Figure 2

Supplementary Figure 3

Uncropped Western blots

## Data Availability

The datasets generated and/or analyzed during the current study are available in the published article. Additional information and materials are available from the corresponding author upon reasonable request.

## References

[1] Singer M, Deutschman CS, Seymour CW, Shankar-Hari M, Annane D, Bauer M, et al. The third international consensus definitions for sepsis and septic shock (Sepsis-3). JAMA. 2016;315:801–10. 26903338 10.1001/jama.2016.0287 PMC4968574

[2] Zhang YY, Ning BT. Signaling pathways and intervention therapies in sepsis. Signal Transd Target Ther. 2021;6:407. 10.1038/s41392-021-00816-9 PMC8613465 34824200

[3] Rudd KE, Johnson SC, Agesa KM, Shackelford KA, Tsoi D, Kievlan DR, et al. Global, regional, and national sepsis incidence and mortality, 1990-2017: analysis for the Global Burden of Disease Study. Lancet. 2020;395:200–11. 31954465 10.1016/S0140-6736(19)32989-7 PMC6970225

[4] Raith EP, Udy AA, Bailey M, McGloughlin S, MacIsaac C, Bellomo R, et al. Prognostic accuracy of the SOFA score, SIRS criteria, and qSOFA score for in-hospital mortality among adults with suspected infection admitted to the intensive care unit. JAMA. 2017;317:290–300. 28114553 10.1001/jama.2016.20328

[5] Becker KL, Snider R, Nylen ES. Procalcitonin assay in systemic inflammation, infection, and sepsis: clinical utility and limitations. Crit Care Med. 2008;36:941–52. 18431284 10.1097/CCM.0B013E318165BABB

[6] Song J, Park DW, Moon S, Cho HJ, Park JH, Seok H, et al. Diagnostic and prognostic value of interleukin-6, PENTRAXIN 3, and procalcitonin levels among sepsis and septic shock patients: a prospective controlled study according to the Sepsis-3 definitions. BMC Infect Dis. 2019;19:968. 31718563 10.1186/s12879-019-4618-7 PMC6852730

[7] Pierrakos C, Vincent JL. Sepsis biomarkers: a review. Crit Care. 2010;14:R15. 20144219 10.1186/cc8872 PMC2875530

[8] Hotchkiss RS, Moldawer LL, Opal SM, Reinhart K, Turnbull IR, Vincent JL. Sepsis and septic shock. Nat Rev Dis Primers. 2016;2:16045. 28117397 10.1038/nrdp.2016.45 PMC5538252

[9] Shankar-Hari M, Rubenfeld GD. Understanding long-term outcomes following sepsis: implications and challenges. Curr Infect Dis Rep. 2016;18:37. 27709504 10.1007/s11908-016-0544-7 PMC5052282

[10] van der Poll T, Shankar-Hari M, Wiersinga WJ. The immunology of sepsis. Immunity. 2021;54:2450–64. 34758337 10.1016/j.immuni.2021.10.012

[11] Koths K, Taylor E, Halenbeck R, Casipit C, Wang A. Cloning and characterization of a human Mac-2-binding protein, a new member of the superfamily defined by the macrophage scavenger receptor cysteine-rich domain. J Biol Chem. 1993;268:14245–49. 8390986

[12] Grassadonia A, Tinari N, Iurisci I, Piccolo E, Cumashi A, Innominato P, et al. 90K (Mac-2 BP) and galectins in tumor progression and metastasis. Glycoconj J. 2002;19:551–56. 14758079 10.1023/B:GLYC.0000014085.00706.d4

[13] Liu FT, Stowell SR. The role of galectins in immunity and infection. Nat Rev Immunol. 2023;23:479–94. 36646848 10.1038/s41577-022-00829-7 PMC9842223

[14] Hong CS, Park MR, Sun EG, Choi W, Hwang JE, Bae WK, et al. Gal-3BP negatively regulates NF-κB signaling by inhibiting the activation of TAK1. Front Immunol. 2019;10:1760. 31402917 10.3389/fimmu.2019.01760 PMC6677151

[15] Pesole PL, Liso M, Donghia R, Guerra V, Lippolis A, Mastronardi M, et al. 90K/Mac-2 BP is a new predictive biomarker of response to infliximab therapy in IBD patients. Int J Mol Sci. 2023;24:3955. 36835367 10.3390/ijms24043955 PMC9966915

[16] Peretz ASR, Rasmussen NS, Jacobsen S, Sjöwall C, Nielsen CT. Galectin-3-binding protein is a novel predictor of venous thromboembolism in systemic lupus erythematosus. Clin Exp Rheumatol. 2021;39:1360–68. 33337998 10.55563/clinexprheumatol/ol0vqj

[17] Abo-Elyazeed S, Gheida S, Soliman G, Gamea M. Plasma galectin-3-binding protein level in patients with systemic lupus erythematosus. Tanta Med J. 2018;46:249.

[18] El Bannoudi H, Cornwell M, Luttrell-Williams E, Engel A, Rolling C, Barrett TJ, et al. Platelet LGALS3BP as a mediator of myeloid inflammation in systemic lupus erythematosus. Arthritis Rheumatol. 2023;75:711–22. 36245285 10.1002/art.42382 PMC11934290

[19] Lee PC, Wu CJ, Lee IC, Lee CJ, Hou MC, Huang YH. Serum fibrosis marker M2BPGi-based novel score predicts survival of unresectable HCC undergoing immunotherapy. JHEP Rep. 2025;7:101491. 40837082 10.1016/j.jhepr.2025.101491 PMC12362694

[20] Kim DH, Sung M, Park MS, Sun EG, Yoon S, Yoo KH, et al. Galectin 3-binding protein (LGALS3BP) depletion attenuates hepatic fibrosis by reducing transforming growth factor-β1 (TGF-β1) availability and inhibits hepatocarcinogenesis. Cancer Commun. 2024;44:1106–29. 10.1002/cac2.12600 PMC11483554 39073023

[21] Liu Y, Liang L, Li J, Pang T, Zhang SH, Xia ZY. Aberrant expression of LGALS3BP drives an unfavorable prognosis and more aggressive in HCC via regulating PI3K/AKT signaling. Tissue Cell. 2024;89:102471. 39029315 10.1016/j.tice.2024.102471

[22] Kim SS, Park I, Kim J, Ka NL, Lim GY, Park MY, et al. Secreted LGALS3BP facilitates distant metastasis of breast cancer. Breast Cancer Res. 2025;27:4. 39789641 10.1186/s13058-024-01958-8 PMC11715970

[23] Cho SH, Shim HJ, Park MR, Choi JN, Akanda MR, Hwang JE, et al. Lgals3bp suppresses colon inflammation and tumorigenesis through the downregulation of TAK1-NF-κB signaling. Cell Death Discov. 2021;7:65. 33824294 10.1038/s41420-021-00447-7 PMC8024364

[24] Sung M, Kim DH, Sun EG, Hwang JE, Cho SH, Chung IJ. et al. LGALS3BP induces insulin resistance via TLR2-IKKα/β pathway-mediated IRS1 serine phosphorylation. Endocrinol Metab. 2025;41:121–137. 10.3803/EnM.2025.2448 PMC12963772 41121618

[25] Ji D, Li J, Liu A, Ye R, Zhang S, Gao L, et al. Predictive value of combined detection of serum LGALS3BP and GDF-15 for the prognosis of ICU sepsis patients. Infect Drug Resist. 2024;17:4417–26. 39431211 10.2147/IDR.S468298 PMC11488509

[26] Luo M, Zhang Q, Hu Y, Sun C, Sheng Y, Deng C. LGALS3BP: a potential plasma biomarker associated with diagnosis and prognosis in patients with sepsis. Infect Drug Resist. 2021;14:2863–71. 34335032 10.2147/IDR.S316402 PMC8318715

[27] Bosquillon de Jarcy L, Akbil B, Mhlekude B, Leyens J, Postmus D, Harnisch G, et al. 90K/LGALS3BP expression is upregulated in COVID-19 but may not restrict SARS-CoV-2 infection. Clin Exp Med. 2023;23:3689–3700. 37162650 10.1007/s10238-023-01077-2 PMC10170455

[28] Gutmann C, Takov K, Burnap SA, Singh B, Ali H, Theofilatos K, et al. SARS-CoV-2 RNAemia and proteomic trajectories inform prognostication in COVID-19 patients admitted to intensive care. Nat Commun. 2021;12:3406. 34099652 10.1038/s41467-021-23494-1 PMC8184784

[29] Park M, Hur M, Kim H, Lee CH, Lee JH, Kim HW, et al. Novel usefulness of M2BPGi for predicting severity and clinical outcomes in hospitalized COVID-19 patients. Diagnostics. 2025;15:937. 40218287 10.3390/diagnostics15070937 PMC11989196

[30] Wen R, Liu YP, Tong XX, Zhang TN, Yang N. Molecular mechanisms and functions of pyroptosis in sepsis and sepsis-associated organ dysfunction. Front Cell Infect Microbiol. 2022;12:962139. 35967871 10.3389/fcimb.2022.962139 PMC9372372

[31] Saavedra-Torres, Pinzón-Fernández JS, Ocampo-Posada MV, Nati-Castillo M, Jiménez Hincapie LA HA, Cadrazo-Gil EJ, et al. Inflammasomes and signaling pathways: key mechanisms in the pathophysiology of sepsis. Cells. 2025;14:930. 40558557 10.3390/cells14120930 PMC12191029

[32] Shi J, Zhao Y, Wang K, Shi X, Wang Y, Huang H, et al. Cleavage of GSDMD by inflammatory caspases determines pyroptotic cell death. Nature. 2015;526:660–65. 26375003 10.1038/nature15514

[33] Bai B, Yang Y, Wang Q, Li M, Tian C, Liu Y, et al. NLRP3 inflammasome in endothelial dysfunction. Cell Death Dis. 2020;11:776. 32948742 10.1038/s41419-020-02985-x PMC7501262

[34] Ma Y, Meng J, Sun F, Lei M. Targeting the NLRP3 inflammasome in sepsis: molecular mechanisms and therapeutic strategies. Cytokine Growth Factor Rev. 2025;86:57–70. 41045688 10.1016/j.cytogfr.2025.09.006

[35] Wree A, Eguchi A, McGeough MD, Pena CA, Johnson CD, Canbay A, et al. NLRP3 inflammasome activation results in hepatocyte pyroptosis, liver inflammation, and fibrosis in mice. Hepatology. 2014;59:898–910. 23813842 10.1002/hep.26592 PMC4008151

[36] Wiersinga WJ, van der Poll T. Immunopathophysiology of human sepsis. EBiomedicine. 2022;86:104345. 36470832 10.1016/j.ebiom.2022.104363 PMC9783164

[37] Ren J, Ren J, Pan Y. The expression of serum LGALS3BP, FGF-21, Trx-1 and their relationship with prognosis in patients with sepsis. J Clin Emerg. 2022;23:246–50.

[38] Lee SI, Kim NY, Chung C, Park D, Kang DH, Kim DK, et al. IL-6 and PD-1 antibody blockade combination therapy regulate inflammation and T lymphocyte apoptosis in murine model of sepsis. BMC Immunol. 2025;26:3. 39806304 10.1186/s12865-024-00679-z PMC11731149

[39] Xu S, Pan X, Mao L, Pan H, Xu W, Hu Y, et al. Phospho-Tyr705 of STAT3 is a therapeutic target for sepsis through regulating inflammation and coagulation. Cell Commun Signal. 2020;18:104. 32641132 10.1186/s12964-020-00603-z PMC7341624

