# Supplementary material for: Secretory LGALS3BP exacerbates sepsis-associated liver dysfunction by activating inflammasome-mediated pyroptosis
Source: Cell Death Discov. 2026 Jun 23;12:355. doi: 10.1038/s41420-026-03198-5 (PMC13542268; doi:10.1038/s41420-026-03198-5)
Supplement: Supplementary file 1 [file 41420_2026_3198_MOESM1_ESM.docx]

**Supplementary Information**

**Supplementary Tables**

**Table S1: Primary antibodies used in the study.**

| **Antibody** | **Manufacturer** | **Cat. No.** | **Application** | **Dilution** |
| --- | --- | --- | --- | --- |
| Lgals3bp | IBL | 28125 | WB  IHC  IF  Neutralization | 1:5000  1:2000  1:50  1:40 |
| NLRP3 | CST | 15101 | WB | 1:1000 |
| ASC | CST | 67824 | WB  IF | 1:1000  1:200 |
| Cleaved Caspase1 | CST | 89332 | WB | 1:1000 |
| Caspase1 | CST | 24232 | WB | 1:1000 |
| Caspase1 | Thermo | PA599390 | IHC | 1:2000 |
| GasderminD | CST | 39754 | WB | 1:1000 |
| Cleaved GasderminD | CST | 10137 | WB | 1:1000 |
| Cleaved IL-1beta | CST | 63124 | WB | 1:1000 |
| IL-1β | CST | 31202 | WB  IHC | 1:1000  1:50 |
| IL-18 | CST | 57058 | WB | 1:1000 |
| TLR2 | CST | 13744 | WB | 1:1000 |
| TLR4 | CST | 14358 | WB | 1:1000 |
| Myd88 | CST | 4283 | WB | 1:1000 |
| pIRF3 | CST | 29047 | WB | 1:1000 |
| IRF3 | CST | 4302 | WB | 1:1000 |
| pIRF7 | CST | 24129 | WB | 1:1000 |
| IRF7 | CST | 39659 | WB | 1:1000 |
| pNF-κB | CST | 3033 | WB | 1:1000 |
| NF-κB | CST | 6956 | WB | 1:1000 |
| β-actin | Abcam | ab6276 | WB | 1:5000 |
| β-actin | Abcam | ab8227 | WB | 1:5000 |
| Anti-mouse IgG(H+L) | Jackson ImmunoResearch | 115-035-003 | WB | 1:4000 |
| Anti-rabbit IgG(H+L) | Jackson ImmunoResearch | 111-035-003 | WB | 1:4000 |
| Anti-Rabbit IgG,  Alexa Fluor 488 | Invitrogen | A11034 | IF | 1:500 |

All secondary HRP-conjugated antibodies for western blotting analysis were purchased from Jackson ImmunoResearch Laboratories. Abbreviations: WB, Western blot; IHC, Immunohistochemistry; IF, Immunofluorescence; CST, Cell Signaling Technology.

**Supplementary Figures**

**Supplementary Figure 1. Increased serum LGALS3BP levels and physiological alterations in Lgals3bp KI mice during sepsis.**

(A) Serum LGALS3BP levels were significantly elevated in Lgals3bp knock-in (KI) mice compared with control (CON) mice. (B) Body weight, liver weight, and spleen weight were measured in CON and KI mice following sham or CLP surgery, corresponding to the experiment shown in Figure 2C. Data are presented as mean ± SEM. Statistical analysis was performed using **two-way ANOVA followed by Tukey’s multiple comparisons test**; ns, not significant; **p* < 0.05, ***p* < 0.01.

**Supplementary Figure 2. Transcriptomic enrichment of interleukin, TLR, and NOD signaling pathways in LGALS3BP-overexpressing livers under sepsis.**

(A) Heatmap showing representative differentially expressed genes (DEGs) in Lgals3bp knock-in (KI) and control (CON) mouse livers under both sham and cecal ligation and puncture (CLP) conditions. Hepatic *Lgals3bp* overexpression, in combination with CLP-induced septic stress, led to a distinct transcriptional reprogramming of the liver. (B) Enrichment plots for the top ten gene sets enriched in Gene Set Enrichment Analysis (GSEA) Hallmark analysis. These plots show the running enrichment score (ES) profile and the distribution of gene set members along the rank-ordered gene list. In each graph, the probes on the far left (red) represent genes most correlated with upregulation, while those on the far right (blue) indicate genes most correlated with downregulation in the Lgals3bp-overexpressing group. The green line represents the running ES across the gene list. The normalized enrichment score (NES) and false discovery rate (FDR) are provided for each gene set. These plots indicate significant enrichment of interleukin-1, interleukin-17, MAP kinase activation, TLR cascades (TLR1/2/6/9), MYD-88, and TRIF-mediated TLR4 signaling, and NOD signaling pathways in KI livers compared with CON following CLP induction. These enriched pathways highlight enhanced inflammatory, innate immune, and injury–related responses driven by hepatic LGALS3BP overexpression during sepsis.

**Supplementary Figure 3. Increased secretion of LGALS3BP and enhanced pyroptosis in hepatocytes.**

(A) Basal LGALS3BP secretion levels were quantified in culture supernatants of Lgals3bp knock-in (KI) and control (CON) hepatocytes, confirming enhanced extracellular release of LGALS3BP in KI cells. (B) Representative immunofluorescence images of primary hepatocytes stained with PI (red) and DAPI (blue), showing increased PI-positive cells in rLGALS3BP-treated hepatocytes under LPS + NiG stimulation. Quantification confirmed a significant increase in PI-positive pyroptotic cells (scale bar, 20 μm). Data are presented as mean ± SEM. ****p* < 0.001, *****p* < 0.0001.
