# Supplementary material for: Secretory LGALS3BP exacerbates sepsis-associated liver dysfunction by activating inflammasome-mediated pyroptosis
Source: Cell Death Discov. 2026 Jun 23;12:355. doi: 10.1038/s41420-026-03198-5 (PMC13542268; doi:10.1038/s41420-026-03198-5)
Supplement: Supplementary file 2 [file 41420_2026_3198_MOESM5_ESM.docx]

**Supplementary Information**

**Full-length, uncropped original Western blots**
